# Editorial: Genetic association studies in oil seeds

**DOI:** 10.3389/fgene.2024.1411718

**Published:** 2024-05-08

**Authors:** V. G. Shobhana, Silvas J. P. Kirubakaran, Song Li, T. P. Parthiban

**Affiliations:** ^1^ Department of Plant Molecular Biology and Biotechnology, Centre for Plant Molecular Biology, Tamil Nadu Agricultural University, Coimbatore, India; ^2^ Upstream Biotechnology, Durham, NC, United States; ^3^ Joint International Research Laboratory of Agriculture and Agri-Product Safety, Yangzhou University, Yangzhou, China; ^4^ International Rice Research Institute, Manila, Philippines

**Keywords:** oilseeds, genetic association, yield, quality, QTL

Crop improvement traits that are significant for agriculture are genetically complex and regulated by polygenes. These polygenes are spread across crop genomes and mapped as genomic targets known as quantitative trait loci (QTLs). Genome-Wide Association (GWA) mapping is an effective plant breeding strategy for detecting natural allelic variations and associating haplotype polymorphisms with valuable agronomic traits such as yield, (a) biotic resistance, and nutritional quality traits. GWA has gained momentum over traditional mapping by documenting alleles/QTLs with a higher resolution by addressing the population structure and linkage disequilibrium (LD). The success of GWA relies on the germplasm choice, population size and diversity, molecular marker density, accurate phenotypic data, and appropriate statistical analyses. The biological value of the genomic regions identified by QTL or GWA warrants validation through diverse functional genomic approaches that drive crop improvement in commercial crops. Combining mapping and functional genomic strategies will enhance the use of genetic variation to improve economically valuable traits of crop plants. Association mapping studies in oilseed crops are at an early stage and are accelerating at a faster pace. Association mapping would unquestionably find genomic solutions to mitigate losses caused by both biotic and abiotic factors, with the success of identifying true associations depending on the marker with higher association signals and their positions within LD.

This editorial embarks on discoveries made with association mapping of the complex traits of economically valuable oilseed crops. Soybean is an important legume and its oil is used in major industrial and pharmaceutical applications and biodiesel production. Lipoxygenases (LOXs) are a family of iron or manganese-containing dioxygenases that catalyze the oxygenation of polyunsaturated fatty acids in plants. Lipoxygenase (LOXs) genes are known to play pivotal roles in regulating growth and development, and orchestrate plant stress responses. The study done by Zhang et al. investigated the phylogenetic divergence of LOX genes in the genome of the Chinese elite cultivar, Zhonghuang 13 and their evolutionary history over domestication provides insights for optimizing stress response, growth and development, hormone response, and light response in soybean. Further mining of LOX variants in wild soybean progenitors revealed the role of genome events, such as duplication and translocation of segments between chromosomes for LOX genes. Structural analysis, coupled with tissue-specific expression analysis, provided insights into conserved motifs and domains, offering clues about their functional roles.

Agricultural research is undergoing a transformative shift through the integration of genomic mapping tools with genomic selection (GS) technologies to accelerate genetic gain for agronomically valuable traits in crop breeding pipelines. Safflower is a global multipurpose crop with seed oil preferred for its high oleic and linoleic acid contents. This study employed a GS approach to simultaneously improve traits with low heritability, such as grain yield, plant height, and days to flowering. Zhao et al. meticulously improved the selection accuracy of grain yield in safflower along with plant height measurements. This study deployed state-of-the-art methods, including genotype × environment interactions in the GS approach, and enhanced the selection accuracy and genetic prediction accuracy for grain yield and oil content.

The aim was to unravel the genomic regions underpinning seed size and shape, which are the key determinants of soybean seed yield. Jiang et al. deployed Quantitative Trait Loci (QTL) mapping to identify genomic regions controlling seed size related attributes across environments. The results revealed a complex genomic landscape with multiple QTLs exerting an influence on seed weight, length, width, and length-to-width ratio. Armed with information on genomic regions associated with multiple seed-related traits, breeders can accelerate the development of improved soybean varieties. Genomic analysis and functional annotation of candidate genes underlying seed size QTLs offer the potential to regulate soybean seed size traits efficiently through functional genomics approaches.

Similar to soybean, sesame is another crop that is gaining worldwide recognition for its healthy functional ingredients in seed oil. Lignans and fat-soluble and water-soluble compounds were the predominant components of sesame oil. Kim et al. conducted a QTL-seq analysis by pooling individuals with contrasting lignan content phenotypes and identified specific genomic regions with interaction ability within the genome. The identification of genomic regions associated with lignan content lays the foundation for breeding resilient and highly nutritive sesame varieties.

Although research progress on understanding the genomes of woody oilseed plants lags behind herbaceous oil crops, the technological development of sequencing technologies and reduced costs have enabled progress in the sequencing of woody oilseed plants, which are deciduous under shrubs or small trees that are native Chinese woody species that can survive in a wide range of growing environments. Yellow horn fruit is rich in unsaturated fatty acids and is a source of high-grade vegetable oils with nutritive value and health benefits. In addition to unsaturated fatty acids (linoleic acid and oleic acid), a minor percentage content of a relatively rare fatty acid, nervonic acid, is indispensable for nervous system development, making it more desirable. Agronomic and physiological studies of yellow horn fruit coupled with transcriptional analysis by Liu et al. have provided insights into the unique expression patterns of fatty acid biosynthesis pathway genes across different developmental stages of seed coat and kernel development. Gene network analysis provides insights into the complex genetic interactions within the genome during fatty acid biosynthesis. Understanding the diversity and functions of the regulatory mechanisms of the fatty acid biosynthesis pathway allows researchers to fortify the fatty acid and lipid content of woody oil seed plants.

